# Risk factors for superficial digital flexor tendinopathy in Thoroughbred racehorses in South Korea (2015–2019)

**DOI:** 10.1111/evj.14493

**Published:** 2025-03-19

**Authors:** Yungi Choi, Tim Parkin

**Affiliations:** ^1^ Veterinary Department Korea Racing Authority Gwacheon‐si South Korea; ^2^ Bristol Veterinary School University of Bristol Langford UK

**Keywords:** horse, risk factors, superficial digital flexor tendinopathy, Thoroughbred racehorses

## Abstract

**Background:**

Superficial digital flexor (SDF) tendinopathy is one of the most common musculoskeletal diseases in Thoroughbreds and a major cause of retirement from racing among racehorses in South Korea. However, there are no previous epidemiological studies on SDF tendinopathy‐related risk factors for racehorses in South Korea. The interventions derived from such a study could reduce the occurrence of SDF tendinopathy and, therefore, extend horses' racing careers.

**Objectives:**

To identify the risk factors associated with SDF tendinopathy for Thoroughbreds in South Korea.

**Study Design:**

Retrospective case–control study (2015–2019).

**Methods:**

A total of 101 Thoroughbreds that were diagnosed with SDF tendinopathy following a recorded fast‐exercise (training gallop, trial racing, racing) between 2015 and 2019 were included in the study. Additionally, 304 healthy Thoroughbreds (319 events) with at least one recorded fast‐exercise between 2015 and 2019 were also included as controls. Multivariable logistic regression was used to identify risk factors for SDF tendinopathy.

**Results:**

SDF tendinopathy was significantly more likely to be diagnosed after racing or trial racing than after galloping. Horses with lower grades (6, ungraded) based on racing performance were associated with a higher risk of SDFT injuries. An increased risk of SDFT injury was observed with fewer gallop training days in the previous 60 days to the last fast‐exercise, more canter training days in the previous 180 days, or having a period of no fast‐exercise for over 90 days in the year prior to the event.

**Main Limitations:**

Information on the distance and speed of gallop training was not available.

**Conclusions:**

The implementation of risk profiling and other preventive measures for horses with fewer gallop training days before racing could help minimise the number of horses sustaining SDF tendinopathy.

## 1 INTRODUCTION

Thoroughbred racehorses frequently suffer injuries to the Superficial Digital Flexor Tendon (SDFT).^1^, ^2^, ^3^, ^4^ Retirement associated with SDFT injuries in Hong Kong had a 3.2% mean annual cumulative incidence, and the prevalence of SDFT injuries in Japan was reported to be 11%.^1^, ^5^ In South Korea, 25.2% of fatal injuries occurring during racing were associated with SDFT injuries.^6^

Although various risk factors for SDF tendinopathy have been identified in multiple studies,^7^, ^8^, ^9^, ^10^, ^11^, ^12^ there is a large variation in risk factors based on the horse's details,^1^, ^8^, ^9^, ^11^ training regimens,^10^ racecourse,^11^ track surface^11^ and season.^9^, ^11^ To date, there have been no prior epidemiological investigations on risk factors for SDFT injuries for Thoroughbred racehorses in South Korea.

Treatment for SDFT injuries frequently requires a lengthy period of rest, and re‐injury is often reported.^13^, ^14^, ^15^ Horses with an abnormality of SDFT noted on pre‐race inspection were associated with an increased risk of SDFT injuries.^7^ This finding suggests that improvement in equine welfare and longevity of racing could be achieved by the identification of risk factors for SDFT alongside pre‐race inspections. While abnormalities of the suspensory ligament during pre‐race inspection also indicate an increased risk of injuries during races, they are typically studied in conjunction with injuries to the proximal sesamoid bone as part of the suspensory apparatus.^7^ Therefore, conducting research on suspensory apparatus injuries requires not only the collection of ultrasound examination data, but also radiographic examination data.

This study aimed to identify the risk factors associated with SDF tendinopathy in Thoroughbred racehorses in South Korea (2015–2019). The primary hypothesis of this study is that the intensity (race vs. training gallops) and frequency of exercise under training and racing conditions are associated with the risk of SDFT injuries.

## 2 MATERIALS AND METHODS

### 2.1 Study population

South Korea has two Thoroughbred racetracks, Seoul Racetrack and Busan Racetrack, and all racehorses are registered by the Korea Racing Authority (KRA), the official regulatory body for racing in South Korea. Racing in South Korea consists entirely of flat racing on sand tracks and takes place throughout the year. Seoul Racetrack accommodates a maximum of 1460 horses, and this study included racehorses that underwent fast‐exercise (training gallop, trial racing, racing) at least once at Seoul Racetrack from January 2015 to December 2019. This period was selected to avoid interruptions to the pattern of racing and training at Seoul Racetrack since 2020, attributed to the COVID‐19 pandemic.

Racehorses can be retired voluntarily at any time without a Veterinary Officer (VO)'s approval or veterinary examination. Therefore, the number of cases included in the current study may be an underestimate of the actual number of cases of SDF tendinopathy over the period of the study. Between 2015 and 2019, a total of 5714 horses underwent gallop training at least once at Seoul Racetrack, of which 257 (4.50%) horses were identified with SDF tendinopathy following fast work exercise.

### 2.2 Selection of cases and controls

All horses racing at Seoul Racetrack, including those in trial races, are required to undergo pre‐race and post‐race physical inspections performed by a VO employed by the KRA. Additionally, if a more thorough examination is deemed necessary by VOs, the racehorses are referred to the KRA equine hospital. A Veterinary Surgeon (VS) is also present during racetrack training to provide emergency care if needed.

Outside of racing or trial racing, and in the absence of fatal injuries during training, trainers autonomously decide to take their horses to either the KRA equine hospital or a private equine hospital within the racetrack for diagnosis and treatment.

Cases were included if the horses were diagnosed by VSs working at the KRA equine hospital within 3 days following the last date of fast‐exercise, remained in the stables during that time, and had no training on the racetrack between the last fast‐exercise and the diagnosis. A total of 40 horses diagnosed by veterinarians at a private equine hospital at Seoul Racetrack, 102 horses diagnosed more than 3 days after the last date of fast‐exercise or that had trained on the racetrack between the last fast‐exercise and the diagnosis date, and 14 horses with suspected pre‐existing SDF tendinopathy before entering Seoul Racetrack were excluded. Ultimately, 101 horses were included in this study as cases. Only the first occurrence of injury in each horse was eligible for inclusion as a case. The date of the last fast‐exercise event for racehorses prior to the first occurrence of SDF tendinopathy was defined as the date of injury, as we presumed that SDF tendinopathy would mainly occur during galloping rather than cantering, according to a number of studies.^16^, ^17^, ^18^ In this study, a ratio of three controls per case was targeted for the overall study population, although the number of controls slightly exceeded the intended ratio. Case horses were not selected as a control before their SDFT injuries occurred. Additionally, if a horse had multiple dates of fast‐exercise, it could be selected as a control multiple times. A total of 304 horses (319 events) that were not diagnosed with SDF tendinopathy between 2015 and 2019 at Seoul Racetrack were randomly selected as controls. To ensure that the random selection of the control group accurately reflects the underlying population, we compared the distribution of key variables (e.g., age, sex, country of origin) between the control group and the overall population. A total of 405 horses (420 events) were included in this study. Typically, meaningful improvements in statistical power come from choosing 3 or 4 controls per case, whereas higher ratios result in only minimal increases in power.^19^

Injuries were confirmed by a combination of clinical examination and ultrasound examination. The KRA equine hospital serves as a secondary hospital, primarily treating racehorses with severe symptoms. All horses that suffer serious injuries during racing or training on the racetrack are transported by ambulance to the KRA equine hospital located within Seoul Racetrack. Additionally, a diagnosis from VSs at the KRA equine hospital is required for insurance compensation in cases where severe injuries lead to retirement. Therefore, VSs at the KRA equine hospital adhere to specific criteria for diagnosing severe (potentially catastrophic) injuries. Using a previously validated scoring system,^20^ they diagnose SDFT injuries as either severe (potentially catastrophic) or less severe based on the established criteria. Therefore, in this study, the severity of injuries was categorised into severe (potentially catastrophic) and less severe injuries. Three ultrasonographic parameters (lesion echogenicity, proportion of SDFT cross‐sectional area affected maximal injury zone [MIZ‐CSA%], proportion of disruption of the longitudinal fibre pattern [MIZ‐LFP%]) within the MIZ were assessed semi‐quantitatively.

Severe (potentially catastrophic) injury (90 cases) was defined by the following criteria:

- Number of zones affected: 4 or more
- MIZ‐CSA: Greater than 50%
- MIZ‐LFP: Greater than 75%
- Echogenicity: Anechoic
- Lesion type: core lesion

Less severe injury (11 cases) was defined as not meeting the criteria for severe (potentially catastrophic) injuries.

Specifically:

- Three horses: Had core lesions with the number of zones affected being 3 or fewer, anechoic echogenicity, MIZ‐CSA between 25% and 50%, and MIZ‐LFP of 75% or less.
- Eight horses: Did not have core lesions, had the number of zones affected being 3 or fewer, hypoechoic echogenicity, MIZ‐CSA of 25% or less, and MIZ‐LFP of 50% or less.

### 2.3 Risk factors

Potential risk factors were identified from the literature and from a priori hypotheses. The horse's details, types of events, types of riders, the number of canter or gallop training days on the racetrack, the number and distance of races, and other related factors including interaction terms were evaluated. The rider was classified as a jockey or a track rider. Both trial racing and racing are conducted with jockeys. During training on the racetrack, either track riders or jockeys can train the racehorses. Track riders are certified by the KRA to ride and train racehorses during their preparation, requiring a minimum period of riding experience and a certain level of knowledge, but they are not subject to any specific weight restrictions. Each horse's age was documented in years at every start, following the convention that the 1st of January was considered the official birthday for Thoroughbred racehorses in South Korea. There is no compulsory retirement age for Thoroughbred horses in the KRA. The sex of the horse was determined on the date of fast‐exercise and was categorised as intact male, female, or gelding. The country of origin was classified into domestic and foreign horses. In South Korea, racehorses are classified into grades based on their racing performance, ranging from Grade 1 to Grade 6, with Grade 1 representing the highest performance level. Upon passing trial racing, foreign horses are classified as Grade 4, while domestic horses are classified as Grade 6. Horses that do not pass the trial racing are classified as ungraded. All ungraded horses must pass trial racing to be eligible to compete in a race. The KRA runs early training programmes for racehorses between the ages of 18–24 months, with the goal of getting them to make their first start as soon as possible after entering the racetrack. Racehorses imported from overseas and immediately stabled at the racetrack were classified as imported horses, and domestic horses were classified as passed or non‐passed, depending on whether they were used to starting gate procedures and raced for 400 m without interfering with other horses after the starting gate opened. The duration from first entering the stable at Seoul Racetrack to each horse's first race was calculated, regardless of whether the horse left or remained at the stables throughout the duration.

The KRA has recorded whether racehorses train at the gallop or canter on the racetrack since 2014. However, there is no information on the total training distance or the exact speed during training on the racetrack. The number of canter and gallop training days, the number of starts, and the total distance of races were calculated for 1, 2, 3, 6 and 12 months prior to the date of disease occurrence, respectively.^21^ For the duration of 12 months, a distinct group was established, comprising horses that were unable to undergo gallop training on the racetrack or compete in a race not because of other issues but due to their stay outside Seoul Racetrack. The total racing distance covered by the horses, including trial racing, is recorded in metres. Distances for which races were cancelled or for which horses were scratched and did not actually compete were not included.

In the previous 365 days to the last date of fast‐exercise work, we calculated the frequency of periods where there were 60, 90 or 180 days or more without any fast‐exercise work on the racetrack. In South Korea, a similar number of races is held throughout the year regardless of the season, so it is rare for racehorses to have a lay‐up solely due to seasonal factors.

In this study, we also evaluated biologically plausible interaction terms. The first potential interaction term compared the number of canter training days and gallop training days on the racetrack over the same period. The value representing the ratio was adjusted by adding 1, and the higher the value, the smaller the difference between the number of canter training days and gallop training days. The value is calculated using the following formula:

$$\left(\frac{\text{The number of gallop training days}}{\;\text{The number of canter training days}\;}\right)+1.$$

The second potential interaction term compared the number of gallop training days in the previous 60 days to the last date of fast‐exercise to the number of canter training days in the previous 90 days, 180 days and 1 year to the last date of fast‐exercise work. The value representing the ratio was adjusted by adding 1, and the fewer the number of gallop training days in the previous 60 days compared with the number of canter training days in the previous 90, 180 or 365 days, the lower the value. The value is calculated using the following formula:

$$\begin{array}{l} \left(\frac{\text{The number of gallop training days in the previous}\;60\;\text{days}}{\text{The number of canter training days in the previous}\;90\;\text{days,}180\;\text{days,}365\;\text{days}}\right)\;+\;1. \end{array}$$

The third potential interaction term compared the number of gallop training days in the previous 60 days to the last date of fast‐exercise work to the number of gallop training days in the previous 90 days, 180 days and 1 year to the last date of fast‐exercise work. The value representing the ratio was adjusted by adding 1, and the fewer the number of gallop training days in the previous 60 days compared with the number of gallop training days in the previous 90, 180 or 365 days, the lower the value. The value is calculated using the following formula:

$$\begin{array}{l} \left(\frac{\text{The number of gallop training days in the previous}\;60\;\text{days}}{\text{The number of gallop training days in the previous}\;90\;\text{days,}180\;\text{days,}365\;\text{days}}\right)\;+\;1. \end{array}$$

The reason for creating various potential interaction terms based on the number of gallop training days in the previous 60 days is that 40% of the horses had one or fewer of the number of gallop training days in the previous 30 days, which was considered to make it difficult to obtain significant results.

### 2.4 Data analysis

A total of 44 variables for each horse were available for analysis. In our study, all continuous variables were transformed into categorical variables to address potential issues related to the linearity assumption and to prevent multicollinearity. This transformation facilitates more flexible modelling by allowing discrete categorisation, which helps to better meet the assumptions of logistic regression and enhances the stability and interpretability of the model.^22^

Univariable logistic regression was performed to identify potential risk factors from variables that were considered biologically plausible and supported in the literature. Variables with a *p*‐value ≤0.25 were considered for inclusion in the multivariable model built using forward selection. Interaction terms with biological relevance were created and evaluated in the final model.

Multivariable logistic regression was used to mitigate the impact of confounding factors, rather than using a matched design.^22^ Variables were retained in the multivariable model if the likelihood ratio test *p*‐values were <0.05.^22^

The effect of each potential confounder on the estimates for variables in the final model was assessed by adding each one, one at a time, into the final model. If the addition of the potentially confounding variable altered odds ratios for statistically significant variables in the final model by more than 20%,^19^ confounding was considered to be present; the confounder was retained in the final model, and adjusted odds ratios were reported for variables in the final model. The Akaike Information Criterion (AIC) was used to compare competing models, with the model having the lowest AIC being selected as the preferred model.^23^

#### 2.4.1 Statistical analysis software

All statistical analyses were performed using SPSS 26 (IBM Co.) statistical software. Epi‐Info 6 (Centers for Disease Control and Prevention, USA) was used to calculate the statistical power.

#### 2.4.2 Post‐hoc power calculations

With 101 cases and 319 unmatched controls (304 horses) and aiming to identify odds ratios of 2 or more, 80% power is provided (assuming 95% confidence) when the probability of exposure in the control group is between 21% and 55%. Alternatively, if aiming to identify odds ratios of 2.3 or more, 80% power is provided (assuming 95% confidence) when the probability of exposure in the control group is between 11% and 70%.

## 3 RESULTS

### 3.1 Descriptive analysis

The data on Thoroughbred racehorses with SDFT injuries in South Korea between 2015 and 2019 are presented in Table 1. Between 2015 and 2019, a total of 5714 horses underwent gallop training at least once at Seoul Racetrack. The prevalence of SDFT injuries among racehorses at Seoul Racetrack was found to be approximately 1.77%. The prevalence of severe (potentially catastrophic) SDFT injuries was higher during trials or racing (1.10%) compared with gallop training (0.47%). The proportion of horses that sustained severe (potentially catastrophic) SDFT injuries and could not return to racing again was significantly higher (98.89%) compared with those that sustained less severe injuries (54.55%). Among the horses with less severe SDFT injuries, five returned to racing and only two successfully competed in more than five races. However, among those with severe (potentially catastrophic) SDFT injuries, only one horse returned to racing, competing in a single trial race before retirement.

**TABLE 1:** The data on Thoroughbred racehorses with SDFT injuries in South Korea between 2015 and 2019.

Injury classification	No. of horses with SDFT injuries, *n* = 101 (%)	Events		The prevalence during gallop training (%)^a^	The prevalence during trials or racing (%)^a^	The overall prevalence (%)^a^	No. of horses not returning to racing, *n* = 95 (%)	No. of horses raced again [<5 races], *n* = 4 (%)	No. of horses successfully raced again [≥5 races], *n* = 2 (%)
		Gallop training, *n* = 33 (%)	Trials or racing, *n* = 68 (%)						
Severe (potentially catastrophic) injury	90 (89.11)	27 (30.00)	63 (70.00)	0.47	1.10	1.58	89 (98.89)	1 (1.11)	0 (0.00)
Less severe injury	11 (10.89)	6 (54.55)	5 (45.45)	0.11	0.09	0.19	6 (54.55)	3 (27.27)	2 (18.18)

^a^ The prevalence based on a total of 5714 horses that performed fast‐exercise at least once at Seoul Racetrack between 2015 and 2019.

### 3.2 Univariable analysis

Of the 44 variables, 40 with a *p*‐value ≤0.25 identified in the univariable logistic regression were eligible for inclusion in a multivariable model. Table S1 provides the details of these variables.

### 3.3 Multivariable analysis

In the final multivariable model, seven variables were associated with SDF tendinopathy (Table 2). Racing and trialling were associated with 11 times greater odds of diagnosis of SDF tendinopathy than galloping events (95% Confidence Interval [CI] 5.2–25.0; Table 2). Horses ridden by a jockey were associated with 0.35 (95% CI 0.2–0.8) times the odds of SDF tendinopathy compared with horses ridden by a track rider. When the type of riders was excluded as an independent variable in the multivariable analysis, the odds ratio for SDFT injury during trial racing or racing decreased (odds ratio [OR] 6.2; 95% CI 3.6–10.5). Additionally, the relationship with the jockey on SDFT injury risk appeared to reverse, being associated with increased odds in the univariable analysis and with reduced odds in the multivariable analysis. Grade 6 and ungraded racehorses (OR 2.8; 95% CI 1.6–4.9) were more likely to be diagnosed with SDF tendinopathy than Grade 1–5 racehorses. More canter training days in the previous 180 days to the last of fast‐exercise work (OR 1.8; 95% CI 1.1–3.2) and fewer gallop training days in the previous 60 days to the last of fast‐exercise work (OR 1.8; 95% CI 1.1–3.2) were associated with increased odds of SDF tendinopathy. Having a period without fast‐exercise for 90 days or more in the previous 365 days to the last date of fast‐exercise work was associated with increased odds of SDFT injuries (OR 3.1; 95% CI 1.7–5.5). A lower ratio of the number of gallop training days in the previous 60 days to the last date of fast‐exercise work compared with the number of gallop training days in the previous 90 days to the last date of fast‐exercise work (OR 2.2; 95% CI 1.2–3.9) was associated with increased odds of SDF tendinopathy.

**TABLE 2:** Results of multivariable logistic regression model investigating risk factors for SDF tendinopathy in Thoroughbreds at Seoul Racetrack in South Korea (2015–2019).

Risk factors for SDF tendinopathy	Total, *n* = 420	Cases (%), *n* = 101	Controls (%), *n* = 319	*p*‐Value	Odds ratio	95% CI
Event						
Gallop training	266	33 (32.67)	233 (73.04)		1 (REF)	
Trial racing or racing	154	68 (67.33)	86 (26.96)	<0.001	11.389	5.183–25.026
Rider						
Track rider	137	24 (23.76)	113 (35.42)		1 (REF)	
Jockey	283	77 (76.24)	206 (64.58)	0.02	0.352	0.151–0.821
Grade						
1–5	267	51 (50.50)	216 (67.71)		1 (REF)	
6, ungraded	153	50 (49.50)	103 (32.29)	<0.001	2.835	1.639–4.904
No. of canter training days in the previous 180 days to the last date of fast‐exercise work						
≤40	287	56 (55.45)	231 (72.41)		1 (REF)	
≥41	133	45 (44.55)	88 (27.59)	0.03	1.837	1.065–3.168
No. of gallop training days in the previous 60 days to the last date of fast‐exercise work						
3–24	283	52 (51.49)	231 (72.41)		1 (REF)	
≤2	137	49 (48.51)	88 (27.59)	0.03	1.842	1.073–3.162
No. of periods without fast‐exercise for 90 days or more in the previous 365 days to the last date of fast‐exercise work						
0	313	60 (59.41)	253 (79.31)		1 (REF)	
1–2	107	41 (40.59)	66 (20.69)	<0.001	3.098	1.737–5.526
The ratio of the number of gallop training days in the previous 60 days to the last date of fast‐exercise work compared with the number of gallop training days in the previous 90 days to the last date of fast‐exercise work						
>1.6	275	55 (54.46)	220 (68.97)		1 (REF)	
≤1.6	145	46 (45.54)	99 (31.03)	0.008	2.190	1.227–3.910

Abbreviations: CI, confidence interval; REF, reference.

## 4 DISCUSSION

This study aimed to identify the risk factors associated with SDF tendinopathy in South Korea with the objective to help reduce SDFT injuries on racetracks. While there have been numerous studies on risk factors for SDFT injuries in various countries, it may not be suitable to extrapolate these risk factors to racetracks in South Korea because of differences in racing type,^10^, ^15^ track surfaces,^12^, ^13^ or racing season.^24^

In this study, the prevalence of SDFT injuries at Seoul Racetrack in South Korea between 2015 and 2019 was 1.8%. The prevalence of SDFT tendonitis in flat Thoroughbred horses has been reported as 3.2% in Hong Kong,^5^ 11% in Japan.^1^ However, direct comparisons between study results may be inappropriate due to differences in the definitions and diagnostic criteria for SDFT tendinopathy. In this study, the observed prevalence of SDFT injuries was based on horses diagnosed by VSs working at the KRA equine hospital within 3 days following the last date of fast‐exercise, remained in the stables during that time, and had no training on the racetrack between the last fast‐exercise and the diagnosis, which may result in significant differences compared with figures from other countries.

The prognosis of racehorses with SDFT injuries is guarded, and recovery from these injuries often takes a substantial amount of time. It was reported that approximately 20%–60% of injured racing horses could return to racing after healing, but up to 80% of them experience a recurrent injury later on.^14^ In this study, only 5.9% of the total 101 racehorses with SDFT injuries returned to racing, and only 2.0% of the total 101 successfully returned to racing again (≥5 races completed). A study conducted in Hong Kong found that 49%–53.5% of horses that sustained SDFT injuries during racing or training successfully returned to races at least once after the injury, and 31.3%–43.7% of horses raced more than five times after their return.^20^

The observed prognosis of racehorses with SDFT injuries in South Korea appears to be less favourable compared with other countries, such as Hong Kong. However, it is important to note that factors such as insurance compensation policies, racing regulations, and the racing environment influence how SDFT injury cases are identified and selected across countries. Systems in some countries encourage the identification of a wide range of injuries, including mild cases, while in others, only horses with severe injuries that are brought to clinics are included. Furthermore, it might be easier to replace horses in South Korea than in Hong Kong, for example, and as such, the factors that influence the decision to retire a horse in otherwise similar jurisdictions can vary significantly. These differences make it difficult to directly compare the results of studies across different regions. Standardised criteria are needed for more accurate prognostic comparisons between studies.

Identifying risk factors for SDFT injuries and implementing preventive measures in advance are paramount. This study aimed to contribute to the establishment of intervention measures by incorporating 44 variables, with a specific emphasis on including the number of canter and gallop training days and race events among them. However, due to the unavailability of information on the distance and speed of gallop training, caution is advised when interpreting the results of this study.

In the current study, SDF tendinopathy occurred more commonly after trial racing or racing than galloping. This finding was consistent with other studies, which reported that 67% (86/129) of tendon injuries occurred during racing and 19% (25/129) during gallop training.^25^ In Hong Kong, the risk of retirement due to tendon injuries after a barrier trial (race practice) or racing, as the last fast‐paced task prior to retirement, was 10 times higher than that of galloping in normal training.^10^ It is generally accepted that clinical tendinopathy frequently follows degenerative changes in the SDFT matrix, attributed to age and exercise.^26^ However, the occurrence of a serious injury, such as a partial rupture of the SDFT, demands high loads. In vivo, SDFT strain during gallop has been reported to range from 11% to 16%, which closely aligns with the in vitro rupture strains of 12%–21%. These findings suggest that at maximal exercise, SDFT operates close to its physiological limits with a relatively narrow safety margin. Therefore, it is suggested that the risk of severe SDFT injury, serious enough to warrant diagnosis at a secondary care facility like the KRA equine hospital, would have been higher under conditions where greater loads were applied, such as during trial racing or racing, compared with gallop training. It was also reported that tendon injuries were more likely after racing or trial racing than training gallop in Hong Kong. Trial racing may be used to evaluate whether horses have recovered from a previous tendon injury.^10^ This hypothesis was supported by the observation that 46% of horses having trial racing as the last fast‐exercise had undergone a previous veterinary examination or ultrasound examination for tendon injuries.^10^

In this study, when a jockey performed fast‐exercise work on the racehorse, the risk of SDFT injury was reduced by 64.8% compared with when a track rider performed fast‐exercise work. The univariable analysis indicated that the risk of SDFT injury was higher when the horses were ridden by a jockey compared with when they were ridden by a track rider. This highlights the importance of multivariable analysis in adjusting for confounding variables and providing a clearer understanding of the true risk factors. Including the type of riders as a variable in the multivariable analysis allows for the separation of the riders' effect from the overall risk of SDFT injury. The high‐risk environment of racing with jockey riding is reflected more accurately in the adjusted odds ratio.

Research on the risk of injuries to racehorses based on the type of riders is limited. However, it has been reported that there is a higher frequency of fatalities among National Hunt Flat racehorses in the UK when ridden by conditional jockeys compared with when ridden by professional jockeys.^3^ Similarly, horses competing in races limited to non‐professional jockeys were found to have a threefold higher risk of sustaining fatal lateral condylar fractures of McIII/MtIII.^27^ According to another study, it was found that the risk of fatal injuries is higher when racehorses are ridden by a new jockey for the first time, compared with when they are ridden by the same jockey.^28^ In this study, two potential reasons might explain the higher risk of SDFT injury when horses are ridden by track riders compared with when they are ridden by jockeys. First, track riders typically weigh more than jockeys, which increases the load on the racehorse during fast‐exercise work and may elevate the risk of SDFT injury. Although no studies have specifically examined the relationship between jockey weight and the risk of SDFT injury, heavier rider weight could theoretically contribute to tendon strain during high‐speed exercise. Separately, research has shown that horses weighing over 470 kg have a higher risk of developing SDF tendinopathy,^12^ suggesting that greater load on the limbs, whether from a rider or horse weight, could be a contributing factor. Secondly, jockeys usually ride horses closer to race day for training on the racetrack, whereas track riders primarily work with horses that are new to the racetrack, such as two‐year‐olds, or those in the training phase to become race‐ready, including horses returning from lay‐ups. Consequently, only horses that successfully complete training with track riders proceed to jockey‐led sessions for racing. This may contribute to the ‘healthy horse effect’, as only healthy horses advance to this stage.

This study demonstrates that Grade 6 or ungraded horses have higher odds of SDFT injuries compared with horses in Grade 1–5. This finding supports the notion that the preparation phase for racing, as well as the early stages of their careers as racehorses, is a critical period for the risk of SDFT injury. Research has reported that horses were more likely to suffer SDFT injury during training preparation phases that did not involve any starts in official trials or races compared with those preparations that included more than one start.^9^ A study has indicated that horses with official ratings in the top quartile are less likely to be associated with SDF tendinopathy.^11^ It has also been suggested that horses susceptible to injury and breakdown are more likely to incur injuries earlier in their careers.^29^ These results suggest the presence of the ‘healthy horse effect’, as only healthy horses are likely to compete in their first race and continue to perform well, leading to higher ratings. While no specific study has directly investigated the effect of early inspections on reducing severe SDFT injuries, our results indicate that continuous monitoring during the early stages of a racehorse's career could aid in the early detection of tendinopathy, thereby reducing the likelihood of severe injuries. This preventive strategy, as supported by our findings and related research, has the potential to improve the welfare of racehorses and facilitate their repurposing for other uses if needed.

In this study, it was found that having more than 41 canter training days in the previous 180 days to the last date of fast‐exercise work, fewer gallop training days in the previous 60 days, and a lower ratio of gallop training days in the previous 60 days compared with the previous 90 days increased the odds of SDFT injuries. Several research findings related to the risk of SDFT injuries based on workloads before injury occurrence have been presented across various countries. In many studies, it has been consistently shown that lower levels of workloads before injuries were associated with a higher risk of SDFT injuries. In a study conducted at the Hong Kong Jockey Club, reduced intensity during the 180 days preceding the last fast‐paced work day was likely to increase the likelihood of SDFT injuries.^10^ Horses with two or more starts in the previous 3 months had a lower risk of SDF tendinopathy than those with fewer starts in work conducted in Great Britain.^30^ It was also found that the increased number of days since the previous race increased the risk of SDF tendinopathy in Japan.^12^ It has been reported that an interval of >60 days between races was associated with SDFT injuries in Kentucky.^7^ Racehorses that had 1–7 starts in the previous 3 months were at a reduced likelihood of SDF tendinopathy compared with racehorses having no starts or >7 starts in the UK.^11^ Consistent with previous findings, our study revealed that horses with fewer gallop training days in the previous 2 months to the last date of fast‐exercise work were at increased odds of SDFT injuries. Additionally, it was found that the odds of SDFT injuries were higher when the ratio of gallop training days in the previous 60 days to the last date of fast‐exercise work was lower compared with the previous 90 days, indicating fewer gallop training days in the last 60 days. However, it is unlikely that reduced gallop training directly contributes to the risk of developing SDF tendinopathy. Instead, the decrease in gallop training days preceding SDF tendinopathy may be attributed to pre‐existing pathologies or lesions, leading trainers to adjust fast‐exercise regimens. Therefore, by closely monitoring horses whose gallop training decreases compared with their previous training intensity as they approach opportunities where they need to demonstrate their racing performances, such as trial racing or racing, it may be possible to reduce the occurrence of SDFT injuries.

This study also found that the number of periods without fast‐exercise for 90 days or more in the previous 365 days to the last date of fast‐exercise work increased the odds of SDFT injuries. Racehorses may have extended periods without fast‐exercise either as a part of their training regimens, involving voluntary low‐intensity training, or involuntarily due to disease. In this study, the lack of information on why racehorses had no fast‐exercise for over 90 days poses a limitation to accurately interpreting the results. However, it was possible that some of these horses were without fast‐exercise for over 90 days due to a health‐related abnormality. Therefore, it is suggested that horses with mild SDF tendinopathy that had been without fast‐exercise for an extended period may have been more prone to developing severe SDFT injuries when they competed in trial racing or racing. This is because minor disruptions in the tendon matrix composition and arrangement can significantly increase the risk of tendonitis.^31^, ^32^ Other studies have reported that SDFT injuries in the forelimb were 5.5 to 13.5 times more likely in horses assessed to be at an increased risk of injury by regulatory veterinarians, based on pre‐race inspection results, compared with those horses not considered to be at an increased risk of injury.^7^ Furthermore, in research carried out in Japan, an increased cross‐sectional area (CSA) and peritendinous oedema observed in ultrasound examinations were reported to be associated with a higher likelihood of suffering a subsequent severe tendon injury.^33^ Since severe tendon injuries are closely related to previous degeneration, it is crucial to diagnose them preemptively through veterinary examinations to prevent the progression of the condition to a severe stage. Therefore, implementing veterinary examinations for horses that have not undergone fast‐exercise for an extended period could potentially contribute to reducing SDFT injuries.

This study was limited in its ability to identify the extent to which excessive high‐speed exercise, accumulated over a short period, is associated with SDFT injuries. To better understand the relationship between the risk of SDFT injuries and excessive workload over a short period, it would be beneficial to obtain accurate information on the workload of racehorses and conduct regular ultrasound examinations. According to previous research, performing excessive high‐speed exercise for a period of 2 months has been shown to increase the risk of musculoskeletal disease.^34^ It is presumed that SDFT injuries may also occur due to excessive exercise over a short duration. Therefore, further research is necessary in this area.

## 5 CONCLUSIONS

This study identified that the risk of SDFT injuries was higher during trial racing or racing compared with gallop training and that racehorses with decreased training intensity or no fast‐exercise work for over 90 days before the last fast‐exercise session were at greater risk. Therefore, it is believed that there are several factors that could help reduce the risk of severe SDFT injuries in South Korean racing. However, in order to develop training programmes aimed at preventing the disease, further research is needed after gathering more detailed information on the workload associated with fast‐exercise.

## FUNDING INFORMATION

Not applicable.

## CONFLICT OF INTEREST STATEMENT

The authors declare no conflicts of interest.

## AUTHOR CONTRIBUTIONS

**Yungi Choi:** Writing – original draft; methodology; conceptualization; data curation; investigation; validation; formal analysis; resources. **Tim Parkin:** Writing – review and editing; methodology; conceptualization; investigation; validation; supervision.

## DATA INTEGRITY STATEMENT

Yungi Choi had full access to all data in the study and takes responsibility for the integrity of the data.

## ETHICAL ANIMAL RESEARCH

No ethical approval needed for this retrospective study.

## INFORMED CONSENT

Not applicable.

## Supporting information


**Table S1:** Results of univariable logistic regression model investigating risk factors for SDF tendinopathy in Thoroughbreds at Seoul Racetrack in South Korea (2015–2019).

## Data Availability

The data that support the findings of this study are available from Korea Racing Authority. Restrictions apply to the availability of these data, which were used under license for this study.
