# Supplementary material for: Risk factors for superficial digital flexor tendinopathy in Thoroughbred racehorses in South Korea (2015–2019)
Source: Equine Vet J. 2025 Mar 19;58(1):31–9. doi: 10.1111/evj.14493 (PMC12699112; doi:10.1111/evj.14493)
Supplement: Supplementary file 1 — Table S1: Results of univariable logistic regression model investigating risk factors for SDF tendinopathy in Thoroughbreds at Seoul Racetrack in South Korea (2015–2019). [file EVJ-58-31-s001.pdf]

Equine Veterinary Journal BEVA

Table S1: Results of univariable logistic regression model investigating risk factors for SDF tendinopathy in Thoroughbreds at Seoul Racetrack in South Korea (2015–2019).

Risk factors for SDF tendinopathy	Total n=420	Cases (%) n=101	Controls (%) n=319	*p*-value	Odds ratio	95% CI
**Event**						
Gallop training	266	33(32.67)	233(73.04)		1(REF)	
Trial racing or Racing	154	68(67.33)	86(26.96)	<0.001	5.583	3.441-9.057
**Year**						
2015	81	11(10.89)	70(21.94)		1(REF)	
2016	69	14(13.86)	55(17.24)	0.274	1.620	0.682-3.847
2017	86	22(21.78)	64(20.06)	0.055	2.187	0.984-4.865
2018	101	26(25.74)	75(23.51)	0.046	2.206	1.015-4.796
2019	83	28(27.72)	55(17.24)	0.003	3.240	1.482-7.080
**Season**						
Winter (12-2)	91	16(15.84)	75(23.51)		1(REF)	
Spring (3-5)	90	22(21.78)	68(21.32)	0.259	1.517	0.736-3.124
Summer (6-8)	90	27(26.73)	63(19.75)	0.052	2.009	0.994-4.059
Autumn (9-11)	149	36(35.64)	113(35.42)	0.232	1.493	0.774-2.881
**Rider**						
Track rider	137	24(23.76)	113(35.42)		1(REF)	
Jockey	283	77(76.24)	206(64.58)	0.031	1.760	1.054-2.938
**Sex**						
Gelding	136	24(23.76)	112(35.11)		1(REF)	
Intact male	128	34(33.66)	94(29.47)	0.082	1.688	0.936-3.045
Female	156	43(42.57)	113(35.42)	0.046	1.776	1.011-3.120
**Age**						
2	67	9(8.91)	58(18.18)		1(REF)	
3	166	46(45.54)	120(37.62)	0.023	2.470	1.132-5.390
4	113	29(28.71)	84(26.33)	0.056	2.225	0.981-5.048
5≤	74	17(16.83)	57(17.87)	0.149	1.922	0.792-4.665
**Grade**						
1-3	99	20(19.80)	79(24.76)		1(REF)	
4, 5	168	31(30.69)	137(42.95)	0.725	0.894	0.478-1.673
6, ungraded	153	50(49.5)	103(32.29)	0.032	1.917	1.057-3.479
**Country of origin**						
Other countries	89	15(14.85)	74(23.20)		1(REF)	
Korea	331	86(85.15)	245(76.80)	0.076	1.732	0.944-3.177
**Early training program**						
Imported	89	15(14.85)	74(23.20)		1(REF)	
Pass	166	49(48.51)	117(36.68)	0.028	2.066	1.081-3.948
No	165	37(36.63)	128(40.13)	0.295	1.426	0.734-2.772
**No. of days from first entering Seoul Racetrack to their first race**						
≤ 85 days	108	23(22.77)	85(26.65)		1(REF)	
86-113 days	99	21(20.79)	78(24.45)	0.988	0.995	0.511-1.938
114-162 days	102	24(23.76)	78(24.45)	0.698	1.137	0.594-2.177
≥ 163 days	111	33(32.67)	78(24.45)	0.154	1.564	0.846-2.891
**No. of canter training days in the previous 30 days to the last date of fast–exercise work**						
≤ 7	192	35(34.65)	157(49.22)		1(REF)	
8-12	167	49(48.51)	118(36.99)	0.014	1.863	1.135-3.056
≥ 13	61	17(16.83)	44(13.79)	0.107	1.733	0.888-3.383

**No. of canter training days in the previous 60 days to the last date of fast–exercise work**						
≤ 12	181	32(31.68)	149(46.71)		1(REF)	
13-16	95	29(28.71)	66(20.69)	0.016	2.046	1.145-3.654
≥ 17	144	40(39.60)	104(32.60)	0.031	1.791	1.056-3.037
**No. of canter training days in the previous 90 days to the last date of fast–exercise work**						
≤ 17	175	29(28.71)	146(45.77)		1(REF)	
18-23	112	27(26.73)	85(26.65)	0.118	1.599	0.888-2.880
≥ 24	133	45(44.55)	88(27.59)	0.001	2.574	1.506-4.402
**No. of canter training days in the previous 180 days to the last date of fast–exercise work**						
≤ 30	169	31(30.69)	138(43.26)		1(REF)	
31-40	118	25(24.75)	93(29.15)	0.550	1.197	0.664-2.156
≥ 41	133	45(44.55)	88(27.59)	0.002	2.276	1.340-3.867
**No. of canter training days in the previous 365 days to the last date of fast–exercise work**						
≤ 50	164	33(32.67)	131(41.07)		1(REF)	
51-70	118	25(24.75)	93(29.15)	0.827	1.067	0.595-1.913
≥ 71	138	43(42.57)	95(29.78)	0.029	1.797	1.063-3.037
**No. of gallop training days in the previous 30 days to the last date of fast–exercise work**						
≥ 4 days	114	23(22.77)	91(28.53)		1(REF)	
2-3 days	140	29(28.71)	111(34.80)	0.916	1.034	0.560-1.909
0-1 days	166	49(48.51)	117(36.68)	0.080	1.657	0.941-2.919
**No. of gallop training days in the previous 60 days to the last date of fast–exercise work**						
≥ 6 days	135	25(24.75)	110(34.48)		1(REF)	
3-5 days	148	27(26.73)	121(37.93)	0.952	0.982	0.538-1.793
≤ 2 days	137	49(48.51)	88(27.59)	0.002	2.450	1.403-4.278
**No. of gallop training days in the previous 90 days to the last date of fast–exercise work**						
≥ 9 days	125	26(25.74)	99(31.03)		1(REF)	
4-8 days	165	36(35.64)	129(40.44)	0.834	1.063	0.602-1.876
≤ 3 days	130	39(38.61)	91(28.53)	0.093	1.632	0.921-2.892
**No. of gallop training days in the previous 180 days to the last date fast-exercise work**						
≥ 16 days	112	21(20.79)	91(28.53)		1(REF)	
8-15 days	145	36(35.64)	109(34.17)	0.246	1.431	0.781-2.623
≤ 7 days	163	44(43.56)	119(37.30)	0.116	1.602	0.891-2.882
**No. of gallop training days in the previous 365 days to the last date of fast-exercise work**						
≥ 26 days	101	22(21.78)	79(24.76)		1(REF)	
13-25 days	110	23(22.77)	87(27.27)	0.877	0.949	0.491-1.835
≤ 12 days	66	30(29.70)	36(11.29)	0.002	2.992	1.521-5.888
Horses with fewer than 366 days since	143	26(25.74)	117(36.68)	0.486	0.798	0.423-1.507

entering the stable at Seoul Racetrack						
**No. of races in the previous 30 days to the last date of fast-exercise work**						
≥ 1	234	43(42.57)	191(59.87)		1(REF)	
No racing	186	58(57.43)	128(40.13)	0.003	2.013	1.279-3.168
**No. of races in the previous 60 days to the last date of fast-exercise work**						
≥ 2	176	35(34.65)	141(44.20)		1(REF)	
0-1	244	66(65.35)	178(55.80)	0.091	1.494	0.938-2.379
**No. of races in the previous 90 days to the last date of fast–exercise work**						
≥ 3	143	35(34.65)	108(33.86)		1(REF)	
2	152	34(33.66)	118(36.99)	0.669	0.889	0.518-1.525
0-1	125	32(31.68)	93(29.15)	0.832	1.062	0.610-1.847
**No. of races in the previous 180 days to the last date of fast-exercise work**						
≥ 5	177	39(38.61)	138(43.26)		1(REF)	
3-4	148	39(38.61)	109(34.17)	0.365	1.266	0.760-2.108
0-2	95	23(22.77)	72(22.57)	0.683	1.130	0.627-2.037
**No. of races in the previous 365 days to the last date of fast-exercise work**						
≥ 10	99	19(18.81)	80(25.08)		1(REF)	
7-9	114	27(26.73)	87(27.27)	0.427	1.307	0.675-2.530
0-6	64	29(28.71)	35(10.97)	<0.001	3.489	1.729-7.038
Horses with fewer than 366 days since entering the stable at Seoul Racetrack	143	26(25.74)	117(36.68)	0.843	0.936	0.485-1.804
**The total distance of races in the previous 30 days to the last date of fast-exercise work**						
≥ 1,300 m	110	19(18.81)	91(28.53)		1(REF)	
1,000-1,200 m	124	24(23.76)	100(31.35)	0.682	1.149	0.591-2.236
No racing	186	58(57.43)	128(40.13)	0.009	2.170	1.211-3.890
**The total distance of races in the previous 60 days to the last date of fast-exercise work**						
≥ 2,400 m	108	20(19.80)	88(27.59)		1(REF)	
1,500-2,300 m	100	23(22.77)	77(24.14)	0.426	1.314	0.671-2.576
≤ 1,400 m	212	58(57.43)	154(48.28)	0.083	1.657	0.936-2.935
**The total distance of races in the previous 90 days to the last date of fast-exercise work**						
≥ 3,000 m	175	40(39.60)	135(42.32)		1(REF)	
2,000-2,900 m	122	29(28.71)	93(29.15)	0.855	1.052	0.610-1.817
≤ 1,900 m	123	32(31.68)	91(28.53)	0.531	1.187	0.695-2.027
**The total distance of races in the previous 180 days to the last date of fast-exercise work**						
≥ 6,100 m	150	31(30.69)	119(37.30)		1(REF)	
4,100-6,000 m	114	30(29.70)	84(26.33)	0.282	1.371	0.772-2.435
≤ 4,000 m	156	40(39.60)	116(36.36)	0.304	1.324	0.776-2.258

**The total distance of races in the previous 365 days to the last date of fast-exercise work**						
≥ 9,100 m	206	43(42.57)	163(51.10)		1(REF)	
5,100-9,000 m	95	32(31.68)	63(19.75)	0.018	1.925	1.120-3.311
≤ 5,000 m	119	26(25.74)	93(29.15)	0.836	1.060	0.612-1.836
**No. of periods without fast-exercise for 60 days or more in the previous 365 days to the last date of fast-exercise work**						
0	243	45(44.55)	198(62.07)		1(REF)	
1	127	36(35.64)	91(28.53)	0.031	1.741	1.052-2.880
2-4	50	20(19.80)	30(9.40)	0.001	2.933	1.529-5.629
**No. of periods without fast-exercise for 90 days or more in the previous 365 days to the last date of fast-exercise work**						
0	313	60(59.41)	253(79.31)		1(REF)	
1-2	107	41(40.59)	66(20.69)	<0.001	2.619	1.620-4.236
**No. of periods without fast-exercise for 180 days or more in the previous 365 days to the last date of fast-exercise work**						
0	384	87(86.14)	297(93.10)		1(REF)	
1	36	14(13.86)	22(6.90)	0.033	2.172	1.067-4.425
**The ratio of the number of gallop training days to the number of canter training days in the previous 30 days to the last date of fast-exercise work**						
> 1.40	131	21(20.79)	110(34.48)		1(REF)	
1.14-1.40	147	34(33.66)	113(35.42)	0.140	1.576	0.862-2.883
< 1.14	142	46(45.54)	96(30.09)	0.002	2.510	1.399-4.502
**The ratio of the number of gallop training days to the number of canter training days in the previous 60 days to the last date of fast-exercise work**						
> 1.40	146	25(24.75)	121(37.93)		1(REF)	
1.21-1.40	108	22(21.78)	86(26.96)	0.510	1.238	0.655-2.339
< 1.21	166	54(53.47)	112(35.11)	0.002	2.334	1.361-4.002
**The ratio of the number of gallop training days to the number of canter training days in the previous 90 days to the last date of fast-exercise work**						
> 1.40	147	24(23.76)	123(38.56)		1(REF)	
1.21-1.40	113	30(29.70)	83(26.02)	0.046	1.852	1.012-3.391
< 1.21	160	47(46.53)	113(35.42)	0.007	2.132	1.225-3.710
**The ratio of the number of gallop training days to the number of canter training days in the previous 180 days to the last date of fast-exercise work**						
> 1.40	142	25(24.75)	117(36.68)		1(REF)	
1.21-1.40	123	29(28.71)	94(29.47)	0.230	1.444	0.793-2.630
< 1.21	155	47(46.53)	108(33.86)	0.011	2.037	1.174-3.534
**The ratio of the number of gallop**						

**training days to the number of canter training days in the previous 365 days to the last date of fast-exercise work**						
> 1.40	131	25(24.75)	106(33.23)		1(REF)	
1.21-1.40	151	29(28.71)	122(38.24)	0.979	1.008	0.556-1.827
< 1.21	138	47(46.53)	91(28.53)	0.006	2.190	1.251-3.835
**The ratio of the number of gallop training days in the previous 60 days to the last date of fast-exercise work compared to the number of canter training days in the previous 90 days to the last date of fast-exercise work**						
> 1.30	135	18(17.82)	117(36.68)		1(REF)	
1.15-1.30	121	29(28.71)	92(28.84)	0.030	2.049	1.071-3.919
< 1.15	164	54(53.47)	110(34.48)	<0.001	3.191	1.763-5.777
**The ratio of the number of gallop training days in the previous 60 days to the last date of fast-exercise work compared to the number of canter training days in the previous 180 days to the last date of fast-exercise work**						
> 1.14	170	26(25.74)	144(45.14)		1(REF)	
1.09-1.14	82	17(16.83)	65(20.38)	0.284	1.449	0.735-2.853
< 1.09	168	58(57.43)	110(34.48)	<0.001	2.920	1.728-4.936
**The ratio of the number of gallop training days in the previous 60 days to the last date of fast-exercise work compared to the number of canter training days in the previous 365 days to the last date of fast-exercise work**						
> 1.10	148	21(20.79)	127(39.81)		1(REF)	
1.06-1.10	84	18(17.82)	66(20.69)	0.159	1.649	0.822-3.309
< 1.06	188	62(61.39)	126(39.50)	<0.001	2.976	1.712-5.172
**The ratio of the number of gallop training days in the previous 60 days to the last date of fast-exercise work compared to the number of gallop training days in the previous 90 days to the last date of fast-exercise work**						
> 1.80	159	27(26.73)	132(41.38)		1(REF)	
1.61-1.80	116	28(27.72)	88(27.59)	0.178	0.685	0.395-1.188
< 1.61	145	46(45.54)	99(31.03)	0.003	0.440	0.256-0.757
**The ratio of the number of gallop training days in the previous 60 days to the last date of fast-exercise work compared to the number of gallop training days in the previous 180 days to the last date of fast-exercise work**						
> 1.45	170	33(32.67)	137(42.95)		1(REF)	
1.30-1.45	114	21(20.79)	93(29.15)	0.835	0.937	0.511-1.720
< 1.30	136	47(46.53)	89(27.90)	0.003	2.192	1.305-3.684
**The ratio of the number of gallop**						

**training days in the previous 60 days to the last date of fast-exercise work compared to the number of gallop training days in the previous 365 days to the last date of fast-exercise work**						
> 1.30	146	29(28.71)	117(36.68)		1(REF)	
1.20-1.30	86	18(17.82)	68(21.32)	0.845	1.068	0.552-2.066
< 1.20	188	54(53.47)	134(42.01)	0.064	1.626	0.972-2.721

Abbreviations: CI, confidence interval; REF, reference
